# Continuous intravenous lidocaine infusion for postoperative pain and recovery in adults

**DOI:** 10.1007/s10151-018-1890-2

**Published:** 2019-01-21

**Authors:** Hugh M. Paterson

**Affiliations:** Colorectal Surgery, University of Edinburgh, Edinburgh, UK

## Abridged abstract

***Background*** The non-opioid, lidocaine, was investigated in several studies for its use in multimodal management strategies to reduce postoperative pain and enhance recovery. This review was published in 2015 and updated in January 2017.

***Objectives*** To assess the effects (benefits and risks) of perioperative intravenous (IV) lidocaine infusion compared to placebo/no treatment or compared to epidural analgesia on postoperative pain and recovery in adults undergoing various surgical procedures.

***Selection criteria*** We included randomized controlled trials comparing the effect of continuous perioperative IV lidocaine infusion either with placebo, or no treatment, or with thoracic epidural analgesia (TEA) in adults undergoing elective or urgent surgery under general anaesthesia. The IV lidocaine infusion must have been started intraoperatively, prior to incision, and continued at least until the end of surgery.

***Data collection and analysis*** Our primary outcomes were pain score at rest, gastrointestinal recovery and adverse events. Secondary outcomes included postoperative nausea and postoperative opioid consumption.

***Main results*** We included 23 new trials in the update. In total, the review included 68 trials (4525 randomized participants). Trials involved participants undergoing open abdominal (22), laparoscopic abdominal (20), or various other surgical procedures (26). The application scheme of systemic lidocaine strongly varies between the studies related to both dose (1–5 mg/kg/h) and termination of the infusion (from the end of surgery until several days after).

***IV lidocaine compared to placebo*** We are uncertain whether IV lidocaine improves postoperative pain compared to placebo or no treatment at early time points (1–4 h) [standardized mean difference (SMD) − 0.50, 95% confidence interval (CI) − 0.72 to − 0.28; 29 studies, 1656 participants; very low quality evidence) after surgery. Due to variation in the standard deviation (SD) in the studies, this would equate to an average pain reduction of between 0.37 and 2.48 cm on a 0–10-cm visual analogue scale. Assuming approximately 1 cm on a 0–10-cm pain scale is clinically meaningful, we ruled out a clinically relevant reduction in pain with lidocaine at intermediate (24 h) (SMD − 0.14, 95% CI − 0.25 to − 0.04; 33 studies, 1847 participants; moderate-quality evidence), and at late time points (48 h) (SMD − 0.11, 95% CI − 0.25 to 0.04; 24 studies, 1404 participants; moderate-quality evidence). Due to variation in the SD in the studies, this would equate to an average pain reduction of between 0.10 and 0.48 cm at 24 h and 0.08 and 0.42 cm at 48 h. In contrast to the original review in 2015, we did not find any significant subgroup differences for different surgical procedures. We are uncertain whether lidocaine reduces the risk of ileus [risk ratio (RR) 0.37, 95% CI 0.15–0.87; four studies, 273 participants], time to first defaecation/bowel movement [mean difference (MD) − 7.92 h, 95% CI − 12.71 to − 3.13; 12 studies, 684 participants], risk of postoperative nausea (overall, i.e. 0 up to 72 h) (RR 0.78, 95% CI 0.67–0.91; 35 studies, 1903 participants), and opioid consumption (overall) (MD − 4.52 mg morphine equivalents, 95% CI − 6.25 to − 2.79; 40 studies, 2201 participants); quality of evidence was very low for all these outcomes.

***IV lidocaine compared to TEA*** The effects of IV lidocaine compared with TEA are unclear [pain at 24 h (MD 1.51, 95% CI − 0.29 to 3.32; two studies, 102 participants), pain at 48 h (MD 0.98, 95% CI − 1.19 to 3.16; two studies, 102 participants), time to first bowel movement (MD − 1.66, 95% CI − 10.88 to 7.56; two studies, 102 participants); all very low quality evidence]. The risk for ileus and for postoperative nausea (overall) is also unclear, as only one small trial assessed these outcomes (very low quality evidence). No trial assessed the outcomes, ‘pain at early time points’ and ‘opioid consumption (overall)’. The effect of IV lidocaine on adverse effects compared to TEA is uncertain (very low quality evidence).

***Authors’ conclusions*** We are uncertain whether IV perioperative lidocaine, when compared to placebo or no treatment, has a beneficial impact on pain scores in the early postoperative phase, and on gastrointestinal recovery, postoperative nausea, and opioid consumption. There is a lack of evidence about the effects of IV lidocaine compared with epidural anaesthesia in terms of the optimal dose and timing (including the duration) of the administration. We identified three ongoing studies, and 18 studies are awaiting classification; the results of the review may change when these studies are published and included in the review.

## Commentary

The concept of using intravenous lidocaine as an adjunct to general anaesthesia is appealing to the colorectal surgeon. The 2015 edition of this Cochrane review concluded that intravenous (IV) lidocaine had a positive impact on pain, gastrointestinal (GI) recovery, length of hospital stay and opioid requirements—impressive benefits for a cheap and easy intervention [1]. However, despite attracting interest, it has never gained widespread uptake. A revised analysis has just been published, adding 23 studies to give a total of 68 trials with 4525 randomized participants [2]. At a glance, the updated abstract suggests that author conclusions have been substantially downgraded, and a busy colorectal surgeon could not be blamed for moving on. However, in addition to new data, the revision includes new analysis techniques, which, while methodologically sound, have not necessarily improved ease of understanding of the article. As ever, the devil is in the detail.

The main caveat is the tremendous heterogeneity of studies included for analysis. There is heterogeneity of operations (laparoscopic and open, abdominal and non-abdominal), of physiological severity (ranging from breast and appendix surgery to open colectomy and prostatectomy), of dose and duration of IV lidocaine infusion (procedure only to over 24 h) and of geographical location/healthcare setting. Furthermore, if ever there was an illustration of what the late Doug Altman was getting at, it is here: small studies, moderate-to-poor methodology and inconsistent reporting of results [3]. You could be forgiven for raising an eyebrow as all of these data are emptied into the meta-analysis black box, and this is a key point in the technical analysis. Quite appropriately, the authors have accounted for study heterogeneity by reporting prediction interval, which in general finds that the dispersion of the true mean effects in the population is far greater than estimated by basic random effects meta-analysis [4]. Hence, although the random effects confidence intervals in this review are suggestive of a beneficial effect of IV lidocaine on various outcomes including early pain scores (SMD − 0.50, 95% CI − 0.72 to − 0.28), postoperative ileus (RR 0.37, 95% CI 0.15–0.87) and time to defecation (SMD − 7.92 h, 95% CI − 12.71 to − 3.13), conclusions are deemed uncertain because “the range of effects that can be expected in future studies (taking existing heterogeneity into account) indicated that lidocaine may not always be beneficial in an individual setting”. This is self-evident: a trial of the analgesic effects of oral paracetamol would be appropriate in the setting of simple headache but not in traumatic femoral shaft fracture. Despite the authors’ caveats, this review provides evidence that IV lidocaine may well have beneficial effects on recovery variables that are of sharp interest to the colorectal surgeon, but perhaps of less interest to breast or spine surgeons. Our own meta-analysis of the existing studies conducted in the setting of colorectal surgery (four open and five laparoscopic) published in this issue supports this contention [5].

Perioperative IV lidocaine infusion is inexpensive, easy to administer and offers the possibility of clinical benefits of particular relevance to colorectal surgery. However, there remains a need for adequately powered and carefully designed studies within optimized perioperative care pathways to quantify that benefit (or definitively refute the outcomes obtained from small, biased, poorly designed studies). To fulfil this objective, two methodological points should be incorporated. First, return of GI function is a key recovery outcome for colorectal surgery that is inadequately described by univariate measures (such as time to first flatus, time to first bowel movement, and episodes of vomiting). Existing composite endpoints (GI-2 and GI-3) of return of gut function have been validated and should be reported [6]. Second, although we know the important elements of optimal perioperative care, researchers need to report compliance with these elements to interpret the effect of interventions. For example, average length of stay in the existing trials of IV lidocaine in laparoscopic colorectal surgery ranged from 3 to 8 days, suggesting considerable differences in perioperative management. Furthermore, the biggest effect size was found in those studies reporting high compliance with enhanced recovery principles and short length of stay. It may be that a benefit was not detected in other studies because it was lost in the ‘noise’ of a suboptimal patient pathway.

In summary, this Cochrane review suggests that perioperative IV lidocaine could be very beneficial to colorectal surgery patients. We need to up our game in conducting robust studies to test it within best practice settings.
